# Epidemiological and Entomological Evaluations after Six Years or More of Mass Drug Administration for Lymphatic Filariasis Elimination in Nigeria

**DOI:** 10.1371/journal.pntd.0001346

**Published:** 2011-10-11

**Authors:** Frank O. Richards, Abel Eigege, Emmanuel S. Miri, Alphonsus Kal, John Umaru, Davou Pam, Lindsay J. Rakers, Yohanna Sambo, Jacob Danboyi, Bako Ibrahim, Solomon E. Adelamo, Gladys Ogah, Danjuma Goshit, O. Kehinde Oyenekan, Els Mathieu, P. Craig Withers, Yisa A. Saka, Jonathan Jiya, Donald R. Hopkins

**Affiliations:** 1 The Carter Center, Atlanta, Georgia, United States of America; 2 The Carter Center, Jos, Nigeria; 3 University of Jos, Plateau State, Jos, Nigeria; 4 Plateau State Ministry of Health, Jos, Nigeria; 5 Nasarawa State Ministry of Health, Lafia, Nigeria; 6 Nigeria Federal Ministry of Health, Abuja, Nigeria; 7 Centers for Disease Control, Atlanta, Georgia, United States of America; Ghana Health Service, Ghana

## Abstract

The current strategy for interrupting transmission of lymphatic filariasis (LF) is annual mass drug administration (MDA), at good coverage, for 6 or more years. We describe our programmatic experience delivering the MDA combination of ivermectin and albendazole in Plateau and Nasarawa states in central Nigeria, where LF is caused by anopheline transmitted *Wuchereria bancrofti*. Baseline LF mapping using rapid blood antigen detection tests showed mean local government area (LGA) prevalence of 23% (range 4–62%). MDA was launched in 2000 and by 2003 had been scaled up to full geographic coverage in all 30 LGAs in the two states; over 26 million cumulative directly observed treatments were provided by community drug distributors over the intervention period. Reported treatment coverage for each round was ≥85% of the treatment eligible population of 3.7 million, although a population-based coverage survey in 2003 showed lower coverage (72.2%; 95% CI 65.5–79.0%). To determine impact on transmission, we monitored three LF infection parameters (microfilaremia, antigenemia, and mosquito infection) in 10 sentinel villages (SVs) serially. The last monitoring was done in 2009, when SVs had been treated for 7–10 years. Microfilaremia in 2009 decreased by 83% from baseline (from 4.9% to 0.8%); antigenemia by 67% (from 21.6% to 7.2%); mosquito infection rate (all larval stages) by 86% (from 3.1% to 0.4%); and mosquito infectivity rate (L3 stages) by 76% (from 1.3% to 0.3%). All changes were statistically significant. Results suggest that LF transmission has been interrupted in 5 of the 10 SVs, based on 2009 finding of microfilaremia ≥1% and/or L3 stages in mosquitoes. Four of the five SVs where transmission persists had baseline antigenemia prevalence of >25%. Longer or additional interventions (e.g., more frequent MDA treatments, insecticidal bed nets) should be considered for ‘hot spots’ where transmission is ongoing.

## Author Summary

Lymphatic filariasis is a mosquito transmitted disease that is best known for causing elephantiasis (grossly swollen legs and genitals). The current strategy for halting lymphatic filariasis in sub Saharan Africa is to establish programs that deliver 6 or more years of annual doses of tablets in community wide treatment programs (called mass drug administration). The tablets are safe, and donated by Merck & Co. and GlaxoSmithKline. We describe a mass drug administration program in central Nigeria that has, since 2000, provided over 23 million cumulative annual treatments to a population of 3.7 million persons. To assess what should be happening generally throughout the program area, lymphatic filariasis infection was monitored in ten ‘sentinel villages.’ In 2009, sentinel village monitoring showed that lymphatic filariasis infection had been reduced between 67–86% compared to levels present when the program began. However, these results were not as good as desired, and suggest that longer or increased efforts are needed beyond 6 years if lymphatic filariasis elimination is to be achieved.

## Introduction

Lymphatic Filariasis (LF) is a mosquito transmitted parasitic infection that in Africa is caused by *Wuchereria bancrofti*. LF, which has no animal reservoir, is largely rural and transmitted by *Anopheles* mosquitoes in West Africa. The adult worms reside in the human lymphatic vessels and cause lymph flow dysfunction that can result in swelling of limbs (lymphedema, elephantiasis) and genital organs (hydrocele), and painful recurrent febrile attacks of acute adenolymphangitis. Microfilariae released by gravid female *W. bancrofti* worms gain access to the blood stream where they circulate at night and are available for the nocturnally feeding mosquitoes. Microfilariae so ingested pass through three larval molts to reach the L3 stage in about 1–2 weeks; L3 are able to infect humans when infectious mosquitoes return to feed again. The L3 develop to adult male and female worms, where they mate in the human lymphatic system and females produce microfilariae, thus completing the life cycle of the parasite [1].

LF is considered by the World Health Organization (WHO) as one of the ‘tool ready’ neglected tropical diseases (NTDs) [2] because LF transmission can be interrupted by safe oral medications that markedly reduce nocturnal microfilaremia, resulting in fewer mosquitoes being infected when they take a blood meal [3], [4], [5], [6], [7]. Three medicines (ivermectin, diethylcarbamazine, and albendazole) are recommended; each has variable lethal affects on the adult worms, so immediate cure of the LF infection is not achieved with a single treatment [8]. WHO recommends annual community-wide mass drug administration (MDA) with 150 ug/kg of ivermectin (Mectizan®, donated for this purpose by Merck) and 400 mg of albendazole (donated by GlaxoSmithKline) for sub Saharan African LF programs [2], [8].

The potential for global LF eradication was first suggested by the International Task Force for Disease Eradication in 1993 [9]. The current WHO endorsed strategy is based on a 1997 World Health Assembly resolution (WHA50.29) to eliminate LF as a public health problem in Africa by 2020 [3], [4], [5], [6], [10]. The strategy is for MDA programs to provide treatment annually, with good coverage, for 6 years [6], [7], [8]. This is based on the assumption that the ivermectin/albendazole combination will interrupt transmission in all epidemiological settings within 6 years of reaching full geographic coverage [6], [8], [11], a contention that some have challenged [12], [13], [14], [15], [16]. WHO has provided a series of evolving guidelines for monitoring and evaluating the coverage and impact of these programs [3], [7], [17], [18]. In order to reach the goal of LF elimination by 2020, African LF elimination programs in particular need to scale up MDA to reach all targeted LF endemic populations in the next few years [6], [7].

The most populous African nation, Nigeria has an estimated population of 150 million persons. The country is comprised of 36 states and a Federal Capital Territory that are further subdivided into 774 local government areas (LGAs). Given wide LF endemicity and the size of its population, Nigeria ranks third among the most LF endemic countries in the world, after India and Indonesia [19]. Accordingly, the goal of global LF elimination cannot be reached without success in Nigeria. Yet MDA scale up for LF to full geographic coverage in Nigeria has lagged [6], [20]. On the other hand, Nigeria has demonstrated the capacity to undertake major and highly successful efforts against two other NTDs, Guinea worm disease [21] and onchocerciasis [20]. The Nigerian onchocerciasis MDA program based on distribution of ivermectin is the largest in the world [22]. Thus, rapid progress could conceivably also be made against LF, using the MDA strategy that has been launched and maintained so successfully for onchocerciasis.

We report on our 12 year effort to eliminate LF in Plateau and Nasarawa states, which was the first LF elimination effort to be launched in Nigeria. The goal of the program was to demonstrate that annual treatment with ivermectin and albendazole on a large scale could interrupt transmission of *W. bancrofti*. Ten sentinel villages (SVs) scattered throughout the two state area were used to measure impact of the MDA program. The two key impact indicators for transmission interruption in these SVs) were a microfilaremia (mf) prevalence <1% and the absence of L3 infective larvae in the vector mosquitoes. WHO transmission interruption criteria require both an mf prevalence <1% in SVs and a population based assessment of LF antigenemia in children. We conducted such a population based assessment in Plateau and Nasarawa states in 2008 and those results will be reported elsewhere (King, in preparation).

## Methods

### Ethics Statement

The program to eliminate LF is a Federal Ministry of Health initiative, but any individual had the right to refuse to take the medicines offered free of charge by the program. The mapping/rapid assessment, nocturnal blood surveys and entomological monitoring procedures were approved by the Emory University Institutional Review Board (protocol numbers 609–97, 153–2001, and 435–2003) and the Plateau and Nasarawa State Ministries of Health. Consent was obtained at three levels: the team obtained permission from the village chief and his council by reading a previously prepared statement with a description of the purpose of program, and risks and benefits of the activity being performed (mapping/rapid assessment by blood filarial antigen detection, nocturnal blood surveys, or entomological monitoring). The statement texts were approved by IRB and the responses to the questions related to consent were ticked off by the team leader, who then signed the form. A similar statement was read during village-wide health education and mobilization sessions for mapping and nocturnal survey activities, and a household consent was obtained for entomological monitoring (likewise responses to consent questions ticked off and signed by team leaders). For individuals tested in mapping or sentinel village surveys, all subjects provided informed consent. While written consent was obtained when participants were literate, oral consent was approved by the Emory IRB because literacy rates are very low in the rural sentinel village areas. Parents/guardians gave consent on behalf of child participants. Written or oral consent was documented on individual laboratory/result forms filled out by team members during mapping or sentinel village exercises.

### Location of the Program

Plateau state (capital city Jos) and Nasarawa state (capital city Lafia) are located in central Nigeria and have an estimated 4.1 million Hausa-speaking residents, 80% of whom live in agricultural villages. Nasarawa state was formed from Plateau state in 1997. Both Christianity and Islam are practiced there. Plateau and Nasarawa states were challenged by sporadic insecurity over the years of the program related to ethnic and religious conflict, and land ownership issues. Administratively, the two states are divided into 30 Local Government Areas (LGAs): 17 in Plateau and 13 in Nasarawa. Each state and each LGA has ministry of health (MOH) structures (State ministry of health—SMOH; LGA ministry of health—LMOH) that provide medical services through tertiary hospitals (located in the capitals), local hospitals and clinics. The LMOH also has community outreach activities.

### History of the Integrated Program with Respect to LF Elimination

The LF initiative in Plateau and Nasarawa states evolved as an integrated NTD effort starting in 1997, which built upon the annual ivermectin MDA platform for onchocerciasis (river blindness) control launched in the early 1990s. The MOH onchocerciasis program was initially assisted by the River Blindness Foundation, and after 1996 by The Carter Center. The Center works directly with and through the SMOH and LMOH structures, as well as in collaboration with the Federal Ministry of Health (FMOH), which has normative oversight of state and local activities.

The 1992 baseline onchocerciasis mapping established that 12 of the 30 LGAs required ivermectin MDA [23], [24], [25]. Ivermectin treatment was launched in 1992 and full geographic coverage achieved in all rural villages in the 12 LGAs in 1993, when about 600,000 treatments were delivered [23], [24]. An integrated MDA program was launched in 1997 with the objective of ‘piggy-backing’ schistosomiasis control (with praziquantel MDA) and LF elimination (with ivermectin/albendazole combination MDA) onto the river blindness ‘platform.’ [26] Prior to adding albendazole to the onchocerciasis MDA program, entomological and epidemiological studies were conducted in 1998–1999. These studies confirmed that the seven years of ivermectin monotherapy for onchocerciasis had not halted LF transmission in the onchocerciasis endemic LGAs [27], [28].

### LF Mapping

LF mapping activities were conducted in a series of surveys from 1998–2000 using various assays for the LF circulating antigen [29]. In 1998 all villages in two LGAs (Pankshin and Akwanga) selected to pilot the LF program were mapped using either the first generation serum-based immunochromatographic tests [ICT card test– AmRad North South Wales, Australia] or the Og4C3 ELISA [TropBio, Australia]. In short, 149 villages were evaluated by testing 4,451 male villagers ≥15 years of age, 22% of whom tested positive for LF antigen, and 12.9% of whom had filarial hydrocele on physical examination. Only 10% of village samples were LF antigen negative. These results were reported in detail [26], [30]. It should be noted that Pankshin and Akwanga were onchocerciasis endemic LGAs that were being treated with ivermectin when the LF surveys took place.

In 1999–2000 mapping was launched throughout the two state area using the WHO Operational Guidelines for Mapping of Bancroftian Filariasis in Africa [31], in which districts (in this case LGAs) were defined as implementation units [17]. The objective of the survey was to determine the need for LF MDA (i.e., the presence of LF transmission) with an approach biased towards finding LF infection. In each of the 30 LGAs, villages were selected by asking local health authorities if they knew of villages where there had been frequent reports of clinical LF (hydrocele and/or lower extremity lymphedema/elephantiasis) that might suggest filariasis was endemic. The names of those villages were written on slips of paper and 1–4 slips for each LGA were drawn from a hat. In each sample village, after obtaining consent from village leaders and individual participants, LF antigen tests were preformed in 50–100 permanent residents, all ≥15 years of age, with the sample equally divided between males and females. Blood samples (100 ul measured by a calibrated capillary tube) were obtained by finger puncture, then transferred to the pad on the whole blood ICT test kit card (initially produced by AmRad ICT, New South Wales, Australia; now produced as ‘NOW®’ ICT Filariasis kits, Inverness Medical Professional Diagnostics 2 Research Way Princeton, NJ 08540) [32]. The test was then run per manufacturer's instructions, with reading timed precisely and positives being read when two pink lines appeared on the card's reagent impregnated filter paper, negatives when one pink line appeared, and indeterminate for any other finding. The LF village antigen prevalence was calculated by dividing the number of positives by the total number of persons examined. Each LGA's prevalence was calculated as the mean of its sample villages' survey means. All LGAs were found to have an antigen prevalence of >1%, which is the threshold for launching an LGA wide MDA program (see Results).

### Mass Drug Administration

In onchocerciasis co-endemic LGAs, local volunteers trained for ivermectin distribution were present at the beginning of the LF program. These volunteers had been trained under the guidelines and financial support of the African Programme for Onchocerciasis Control (APOC), and were known by that program as community directed distributors (CDDs) [33], [34], We retained that designation (e.g., CDD) for the LF program to emphasize the fact that this was an integrated delivery platform for both onchocerciasis and LF . CDDs were trained or retrained annually to distribute the ivermectin and albendazole tablets with a focus on the need to provide health education about LF and (where appropriate) onchocerciasis; to not confuse the ivermectin tablets (white, small, round, unscored) with the albendazole tablets (white, larger, oblong, scored); and to identify, report and, if necessary, to refer persons with adverse events (AEs) occurring within 48 hours of treatment. Ivermectin treatment was the same as that used in onchocerciasis (150 ug/kg) and was dosed by height. A single albendazole tablet (400 mg) was given together with the ivermectin. Children <90 cm height were not treated. Persons appearing very weak or chronically ill were not treated, and women were not treated if they reported being pregnant or nursing a newborn under one week of age [8]. Persons with AEs (usually headache, fever, and/or abdominal pain within 48 hours of MDA) were treated with oral antihistamines and/or analgesics by local health workers, who reported these results to the LMOH supervisors. CDDs and health workers were instructed on how to identify and refer persons with severe or unusual events occurring post MDA to local facilities staffed by nurses or doctors.

CDDs in most instances went house to house within their areas of responsibility to distribute the medicines; the treatment was directly observed, and the process at the village level was usually completed within 1–2 weeks, but state-wide activities began in March and were not completed until December. In urban areas, treatments were done at a central location such as a clinic, hospital, school, church or mosque. LMOH staff members on motorbikes provided by the program supervised treatment activities, and villages were also independently visited in spot checks by SMOH and/or Carter Center personnel.

A central part of the treatment process was the community register, which was kept by each CDD. Each page in the register was dedicated to a single household in the CDD's area of responsibility, and listed all residents by age and gender, starting with the head of the family. Each household page had sufficient space to record 7 rounds of treatment. Individual treatment was directly observed and then immediately recorded in the register. If an individual eligible for treatment was not present at the time of the household visit, the CDD would return later to find and treat that person.

Using the community register, each CDD would work with his/her LMOH supervisor to prepare a summary report form after the MDA was completed. At the time of the register review all unused tablets were collected by the LMOH supervisor. In turn, working with their SMOH supervisors, LMOH staff summarized their LGA treatment figures monthly. These summaries were compiled at the SMOH into a state monthly treatment report. Copies of LGA and state monthly reports were kept at LGA, State and The Carter Center offices. State level reports were shared with responsible officials at the FMOH monthly.

### Health Education

Health education (HE) was conducted by CDDs and LMOH staff during mobilization activities just prior to MDA, and again during MDA. HE aimed to provide information and understanding about LF infection, disease, treatment and possible adverse events, and to encourage treatment eligible persons to take the tablets. HE was guided and illustrated in colorful flip charts, and posters, pamphlets, and calendars with HE messages. Prototypes were developed and tested by Knowledge-Attitudes-Practices (KAP) surveys conducted in 1999 in villages in the pilot LGAs of Akwanga and Pankshin [26]. Based on the KAP results and experiences with the initial MDAs, materials were revised and then reevaluated with focus groups; minor modifications were made before full scale production for the larger campaign. Onchocerciasis HE was provided as before in coendemic LGAs at the same time as LF HE.

### Scaling up

Scaling up LF MDA to full state-wide geographic coverage was done in four phases. Phase 1 pilot (studies as described above) took place in the two LF-onchocerciasis co-endemic LGAs of Pankshin (Plateau state) and Akwanga (Nasarawa state), where combined ivermectin and albendazole MDA was launched in July 2000 [26]. In 2001 (Phase 2) LF MDA expanded to the remaining 10 LF-onchocerciasis co-endemic LGAs, and included the large towns that had previously been untreated under the onchocerciasis program. In 2002, Phase 3 was launched by expanding into all but four of the non onchocerciasis (LF endemic only) LGAs where no MDA had been previously given. Recruitment and training of an entirely new cadre of CDDs presented new challenges to the program that had until then been integrated into the established onchocerciasis MDA platform. Phase 4 achieved full geographic coverage in 2003 by launching MDA in the remaining LGAs and the urban areas. The scale up challenge of Phase 4 was that of assessing and treating the capital cities of Jos and Lafia for the first time [35].

### Treatment Coverage

The estimated population of the two state area is 4.1 million, based on the 1991 National Nigerian census adjusted forward to the year 2000, and the treatment eligible population (e.g., all persons five years of age and above) is estimated to be 90% of the population, or 3.7 million. That figure, deemed the Ultimate Treatment Goal (UTG), would be the crude coverage target when full geographic coverage was reached (e.g, when all communities in the two state area were offered annual treatment by the program) [36]. UTG coverage was calculated by dividing the numbers of persons treated in a given year by the 3.7 million. Satisfactory coverage was arbitrarily established as ≥85% of the UTG.

Treatments administered compared with their respective community denominators (based on the community CDD registers) were determined in 2004 and 2009 across >3,600 village reports. In addition, for each of ten sentinel villages (described in the next section) community registers were used to determine eligible population treatment coverage annually during the entire MDA period.

To confirm reported coverage, a cluster coverage survey was conducted in 2003, the first year full geographic coverage of the MDA program [17]. A 30-cluster design was used with probability proportional to estimated community size, based on data collected by through the CDD's household registers. Ten households per cluster were selected by random walk; in each sampled household a questionnaire was administered to determine MDA compliance by age and gender. The 2003 treatment coverage assessments were also conducted using the same random walk technique in nine of the ten sentinel villages (one sentinel village [Gwamlar] was not surveyed due to insecurity).

### Monitoring the Impact of the Integrated Program in Sentinel Villages

**Figure 1:**
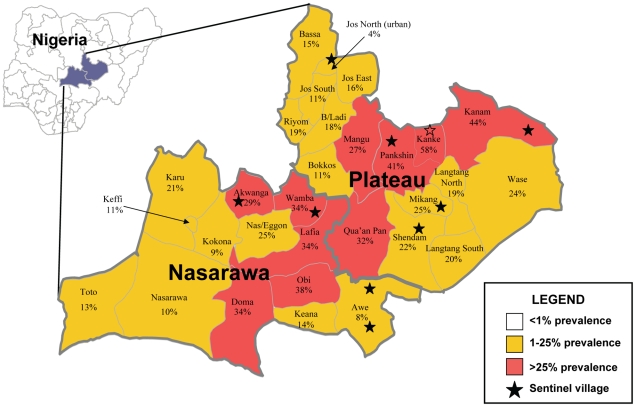
Plateau and Nasarawa States, Nigeria: Baseline LGA LF Antigen prevalence and sentinel village locations. Surveys in 70 villages sampled 6,489 adults for LF antigen in 1999 and 2000 (50–100 adult residents of each village) located in the 30 LGAs comprising Plateau and Nasarawa states. The map is color coded based on the mean antigen results (mean of the village means): shown in red are ten LGAs having a mean baseline antigen prevalence exceeding 25% that form a middle band stretching from southwest to northeast.

Serologic (filarial antigen as determined by ICT test), parasitologic (nocturnal blood slides stained and read for microfilaremia), and entomologic (mosquito dissection for LF larval infection) were the three impact indicators monitored annually in ten sentinel villages (SV). It should be noted that WHO guidelines only require microfilaremia (the ‘key’ indicator) to be monitored in SVs, so our assessment program investigated more parameters than required. SVs were selected based on: 1) being representative of a range of baseline antigen prevalence; 2) being representative of all four phases of the MDA scale up; 3) being representative of both onchocerciasis and non onchocerciasis endemic LGAs; and 4) having village residents and leadership apparently willing to participate in annual nocturnal blood surveys and bimonthly mosquito collections for an indefinite period of time. The location of the SVs is shown by stars in Figure 1, and a list of the ten SVs, with their populations is shown in Table 1.

**Table 1:** Annual reported treatment coverage of eligible population of sentinel villages, and 2003 surveyed coverage.

Sentinel Village	LGA (Baseline Antigen Prev.)	Years of Tx	2009 eligible pop.	2000	2001	2002	2003	2003 (surveyed)	2004	2005	2006	2007	2008	2009
**Akwete**	**Awe (8%)**	**7**	**1123**	NA	NA	NA	77%	92%	94%	90%	77%	100%	94%	98%
**Azara**	**Awe (8%)**	**7**	**7107**	NA	NA	NA	99%	81%	96%	93%	97%	95%	96%	92%
**Babale**	**Jos North (4%)**	**7**	**2394**	NA	NA	NA	89%	67%	90%	90%	87%	90%	91%	0%
**Dokan Tofa**	**Shendan (22%)**	**7**	**2175**	NA	NA	NA	83%	85%	81%	77%	90%	87%	87%	87%
**Gbuwhen**	**Akwanga (29%)** *	**10**	**1221**	78%	72%	50%	100%	98%	100%	99%	100%	98%	100%	88%
**Gwamlar**	**Kanam (44%)** *	**8**	**4917**	NA	NA	92%	81%	NA	79%	75%	90%	89%	91%	91%
**Lankan**	**Pankshin (41%)** *	**10**	**1722**	86%	90%	95%	100%	85%	99%	98%	97%	90%	93%	96%
**Maiganga**	**Wamba (34%)** *	**9**	**350**	NA	100%	100%	95%	90%	100%	100%	100%	100%	88%	100%
**Piapung**	**Mikang (25%)**	**7**	**1680**	NA	NA	NA	85%	71%	81%	89%	96%	90%	90%	90%
**Seri**	**Kanke (58%)** *	**9**	**981**	NA	99%	100%	99%	94%	95%	94%	98%	100%	97%	99%
**TOTAL**			**21276**	85%	96%	92%	90%	82%	92%	89%	94%	92%	93%	92%

Reported treatment of eligible (aged five and above healthy persons) populations in ten sentinel villages (SVs) based on registers kept by community directed volunteer drug distributors (CDDs). A confirmatory coverage survey undertaken in nine of the ten villages in 2003 showed an overall coverage of 82%, lower than the reported coverage that year of 90%. Gwamlar was not surveyed in 2003 due to insecurity. Babale, in the urban area of Jos North, did not get treated in 2009 due to civil unrest, and had the lowest surveyed coverage in 2003.

*Antigen >25%.

NA = Not applicable, in cells corresponding to the years before the MDA program in that SV had been launched.

### Nocturnal Blood Surveys

Nocturnal blood surveys were used to determine SV microfilaremia and antigen prevalence during the same individual blood draw. Surveys were based on convenience samples of permanent village residents and conducted at least 10 months after the last MDA. Each year, one week prior to the survey, the team would obtain permission from the village chief and his council, followed by a village wide HE and mobilization session to explain LF and the purpose of the nightlong survey. At the end of the meeting, persons of all ages were asked to come on the designated night at 9 pm. On the night of the survey, an examination area was set up in a central location and several tables and chairs were placed under a string of light bulbs powered by a gasoline generator. A large TV setup provided continuous video entertainment and gave the survey scene an appealing, festive atmosphere. Guards were present to assure the team and the residents of security during the night. Before beginning, the team again explained the purpose of the survey to those who had gathered. Between 10 pm–2 am, residents aged 2 years and greater presented for examination, and had their age and gender recorded and a finger puncture blood specimen collected by a technician. Parents provided consent on behalf of all child participants. Using disposable calibrated capillary tubes, 100 ul of blood were applied to ICT card tests that were timed and read according to manufacturer's recommendations (see above), and another 60 ul of blood were used to prepare thick blood films. The slides were air dried and returned to the laboratory at Carter Center headquarters in Jos for Giemsa staining and qualitative examination for *W. bancrofti* microfilariae (mf) by trained microscopists. Microfilarial counts were not conducted; slides were read qualitatively (‘positive’ or ‘negative’). Technicians were trained not to confuse mf of *Mansonella perstans* (also prevalent in this part of Nigeria) with those of *W. bancrofti*. All positive slides were confirmed by another microscopist, and 10% of negatives were reread by another microscopist as a standard quality control measure. Another quality control measure was to spend extra time examining blood slides from ICT positive persons. Results were not age adjusted and were expressed as SV antigen prevalence (number ICT positive/number examined X 100) and SV microfilaremia prevalence (number mf slide positive/number examined X 100).

Given the phasing of the MDA, in a given calendar year all SVs were not in the same MDA treatment year. For example, in 2003, the first year of full geographic coverage, SVs in Phase 4 were in their first year of MDA, while SVs in Phase 1 were in their fourth MDA year. In part of our analysis, we corrected for this by grouping SVs based on their MDA treatment round year. In 2009, the last year of monitoring data reported in this study, all villages were being evaluated just prior to 2009 MDA, so that mf prevalence represented a sampling 10 months or more after at least six years of MDA.

‘Baseline’ mf and ICT data calculations for SVs warrant a special comment. In the first years of SV monitoring, the Jos laboratory experienced technical problems with Giemsa stain precipitation. As a result, many slides were ruined and pretreatment mf prevalence could not be established for the five SVs in Phases 1–3. In 2002 the stain problem was solved and we were able to obtain pretreatment mf rates for the five SVs in Phase 4 (Akwete, Azara, Babale, Dokan Tofa, and Piapung). Since mf rates ≥1% were considered indicative of ongoing transmission, and because rates ≥1% were still found in 2002 in Phase 1–3 SVs, we choose to use the post treatment mf data obtained in year 4 of treatment or earlier as the ‘baseline’ mf calculation for those SVs. The exception was Gwamlar, whose first mf data point (also ≥1%) could not be obtained until its sixth year of treatment (2005) due to insecurity in Kanam LGA. Accordingly, it is important to distinguish the term ‘mf pretreatment’ (e.g., prior to MDA) from ‘mf baseline.’ Mf baseline is when the first SV mf values were obtained, and such baseline data could encompass early MDA years. We consider mf baseline as the best term to use considering that all SVs in Phases 1 and 2 were already under ivermectin monotherapy for onchocerciasis, so ‘pretreatment’ would be a misnomer.

Data for the baseline antigen for five villages (Gbuwhen, Gwamlar, Lankan, Maiganga, and Seri) were from the 1999–2000 mapping surveys where only 50–100 adults were sampled. Subsequently larger convenience samples were tested in the nocturnal surveys, including children as young as 2 years of age. In LF endemic areas, adults as a rule have higher antigen prevalence than children, so a spurious drop in prevalence between the mapping baseline and the follow up community samples was expected. In five villages (Akwete, Azara, Babale, Dokan Tofa, Piapung) we combined values from the community wide pre-treatment surveys conducted in 2002 or 2003 with the smaller adult samples obtained in the 1999–2000 mapping surveys. ‘Baseline’ antigen results were compared with results obtained after year 4 of MDA. However, unlike the mf analysis, antigen data obtained between year 1 and 4 of treatment are not included in antigen baseline calculations.

### Entomology

Entomological surveys were conducted every two months in each SV when security conditions allowed. Compounds where the residents agreed to participate were numbered and the even numbered compounds were serially sampled, substituting the odd numbered compounds on occasions when residents in even numbered compounds were not home or could not participate. Collections in odd numbered compounds were also added when numbers of mosquitoes were few during the dry season. Indoor resting mosquitoes were collected in the morning using the pyrethrum knockdown (PK) technique as previously described [28]. The dead and dying mosquitoes that fell onto sheets draped throughout the compound living area were collected by forceps and placed in Petri dishes having moist tissue. Dissections were performed on the day of collection, in the village. Each mosquito was identified as *Anopheles gambiae* sl, *An. funestus*, other *Anopheles* sp, *Culex* sp or ‘other’, and separated into head, thorax and abdomen on a glass slide under a binocular dissecting microscope. Each of these was teased open in a drop of normal saline. The slide preparation was then passed to 100× under a regular microscope where a trained microscopist noted the presence or absence of larval stages (L1–3). Similar to the blood slides, larval stages were recorded qualitatively for any larval stage (positive/negative), and the presence or absence of L3 (positive/negative); numbers of larvae in each stage were not quantified. If more mosquitoes were collected than could be dissected in a day, all *Anophelines* sp mosquitoes were placed in capped tubes with desiccant, labeled, and stored for future molecular testing. Data on each dissected mosquito were entered into a log book and later entered into an Excel file. In the analysis, infected mosquitoes were defined as having any larval stage (L1, L2, or L3). Infectious mosquitoes were defined as those containing L3. Microfilariae found in abdominal examination of blood fed mosquitoes were not considered in the analysis since we were not interested in a xenodiagnostic picture of the inhabitants of the sampled compound where the mosquitoes were collected [37]. Instead, our analysis focused on only *W. bancrofti* larval stages so as to provide an index of community-wide parasite transmission [28]. SV results from the six outings of the year were summed; at least 100 mosquitoes needed to be dissected in a year for that SV result (of that year) to be included in the analysis. ‘Baseline’ mosquito infection rates were aggregate results from ‘pretreatment’ and the first two years of ivermectin/albendazole MDA. The finding of one or more mosquitoes containing infectious larvae (L3) was considered to be indicative of active LF transmission in that SV during the year.

### Statistical Testing

Results of coverage and SV surveys were analyzed in Epi Info 6 (CDC, Atlanta, USA), SAS (SAS institute, Cary, NC), Stata 8.2 (Stata Corporation, College Station, TX) and SUDAAN (Research Triangle Institute, Research Triangle Park, NC). The 2003 treatment coverage survey results and summary SV MDA treatment year aggregate results were expressed with exact binomial 95% confidence intervals. Significance of trends between SV MDA year aggregate values for microfilaremia prevalence, antigen prevalence, and mosquito infection was determined using a Mantel chi-square for trend analysis. Differences between baseline and 2009 microfilaremia prevalence, antigen prevalence, and mosquito infection were compared as categorical data using Pearson chi-square (two tailed) test considering a p-value <0.05 as significant and <0.01 as highly significant. Given the importance of the sixth MDA year in WHO LF elimination policy, statistical testing comparing indices in the sixth MDA year with baseline was also undertaken.

## Results

### LF Mapping

Mapping exercises (Figure 1) to determine LF circulating antigen prevalence were conducted in 1999–2000 in 70 villages in the 30 LGAs. Two villages were sampled from each LGA, with the exception of Toto, Akwanga, Pankshin and Wase (4 villages each); Karu, Jos East, and Shendam (3 villages each); and Keffi and Jos North (1 village each). A total of 6,489 adults were tested, of whom 1,305 (20%) were positive. The mean LGA prevalence (based on the mean of the village means) for the two state area was 23% (range 4–62%). The two states were quite similar, with the Nasarawa mean village prevalence being 21% (range 4–54%) and the Plateau village prevalence mean being 24% (range 4–62%). Figure 1 shows the LGA antigen prevalence stratified by endemicity. The entire two state area was targeted for MDA with ivermectin and albendazole because mapping showed that each LGA implementation unit sampled had an antigen prevalence in adults of >1%. The 30 LGAs were listed in order of descending prevalence and divided into terciles. The upper tercile (10 LGAs) was noted to have an antigen prevalence >25%: these were Akwanga, Doma, Lafia, Obi and Wamba in Nasarawa state, and Kanke, Mangu, Qua'an Pan and Pankshin in Plateau. The spatial distribution of the upper tercile formed an interesting middle band extending from southwest to northeast.

### Mass Drug Administration

**Figure 2:**
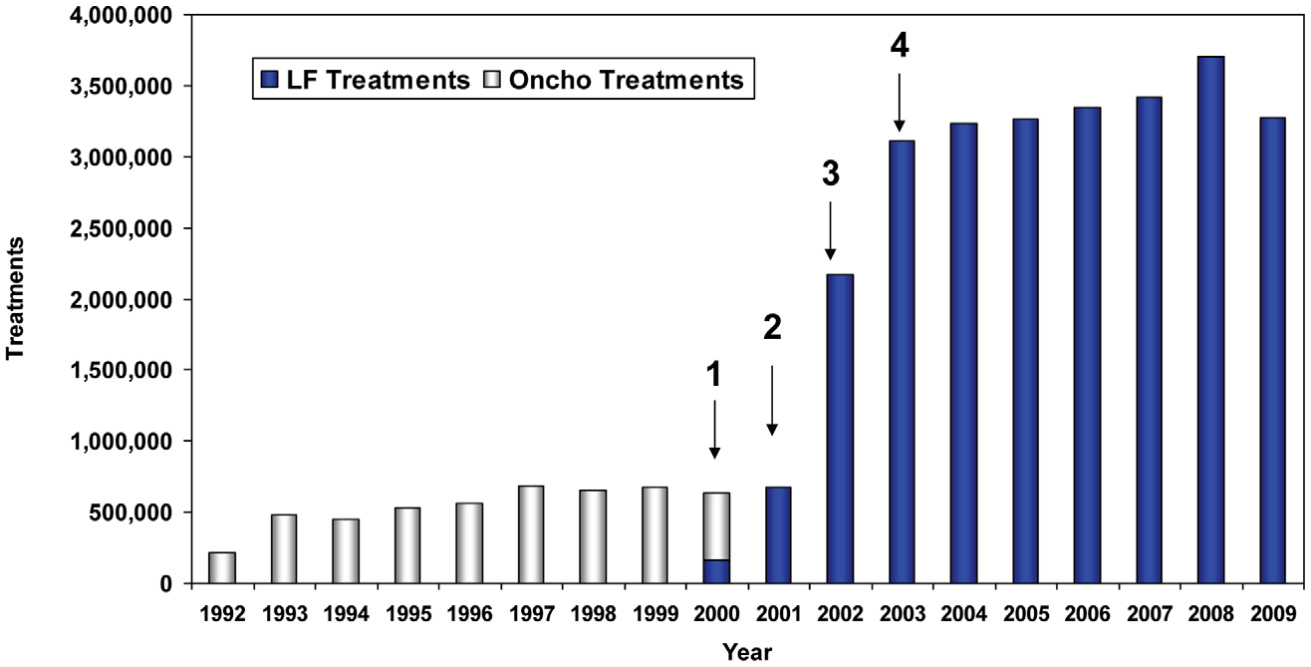
Scaling up onchocerciasis and lymphatic filariasis treatments: 1992–2009. Light bars show ivermectin monotherapy for onchocerciasis MDA provided in rural villages in 12 LGAs deemed onchocerciasis endemic in 1992. Dark bars show LF treatment with ivermectin and albendazole combination therapy. Arrows indicate LF scale-up phases. Phase 1: pilot studies completed in two onchocerciasis endemic LGAs (Pankshin in Plateau state and Akwanga in Nasarawa state). Phase 2: LF MDA expanded to the remaining 10 onchocerciasis endemic LGAs, to now include large towns in those LGAs that had previously been untreated under the onchocerciasis program. MDA in Phases 1 and 2 was for both onchocerciasis control and LF elimination. Phase 3: LF MDA expanded to all but four of the non-onchocerciasis endemic LGAs. Phase 4: LF MDA reached full geographic coverage (all 30 LGAs) and the capital cities of Jos (Plateau) and Lafia (Nasarawa). MDA added in Phases 3 and 4 were only for the purpose of LF elimination.

Figure 2 shows the numbers of treatments provided by the onchocerciasis MDA program (launched in 1992), followed by arrows indicating the phasing in of the LF program (launched in 2000). Expansion into non onchocerciasis endemic areas began in Phase 3, and the LF program reached its full geographic coverage in Phase 4 (that included the major urban areas of Jos and Lafia). Given the magnitude of the scale up required to reach all LF endemic areas, treatments had to be increased 4.6 fold between Phase 2 (when 675,701 treatments were provided) and Phase 4 (when 3,112,379 treatments were provided). Accomplishing this scale up required a three fold increase in CDDs, from 2,424 in the year 2000 to 6,899 by 2003. To maximize coverage and minimize workload, the program continued to recruit and train CDDs annually, training 10,011 in 2009. At that point, each CDD was responsible for treating an average of 347 persons.

Treatment was provided in 8 LGAs for seven years (2003–2009, Figure 2) after full geographic coverage was obtained. However, two LGAs from Phase 1 pilot (Pankshin and Akwanga) were treated for ten years, 10 LGAs from Phase 2 for nine years, and 10 LGAs from Phase 3 for eight years. Note that the Phase 1 and 2 LGAs treated for the longest periods (9–10 years) with combination ivermectin/albendazole also had (from 1992–1999) an additional 8–9 years of ivermectin MDA monotherapy for onchocerciasis. A cumulative total of 26,352,060 combination ivermectin and albendazole treatments were administered in Plateau and Nasarawa states over the ten year LF elimination effort (2000–2009).

### Reported Treatment Coverage

**Figure 3:**
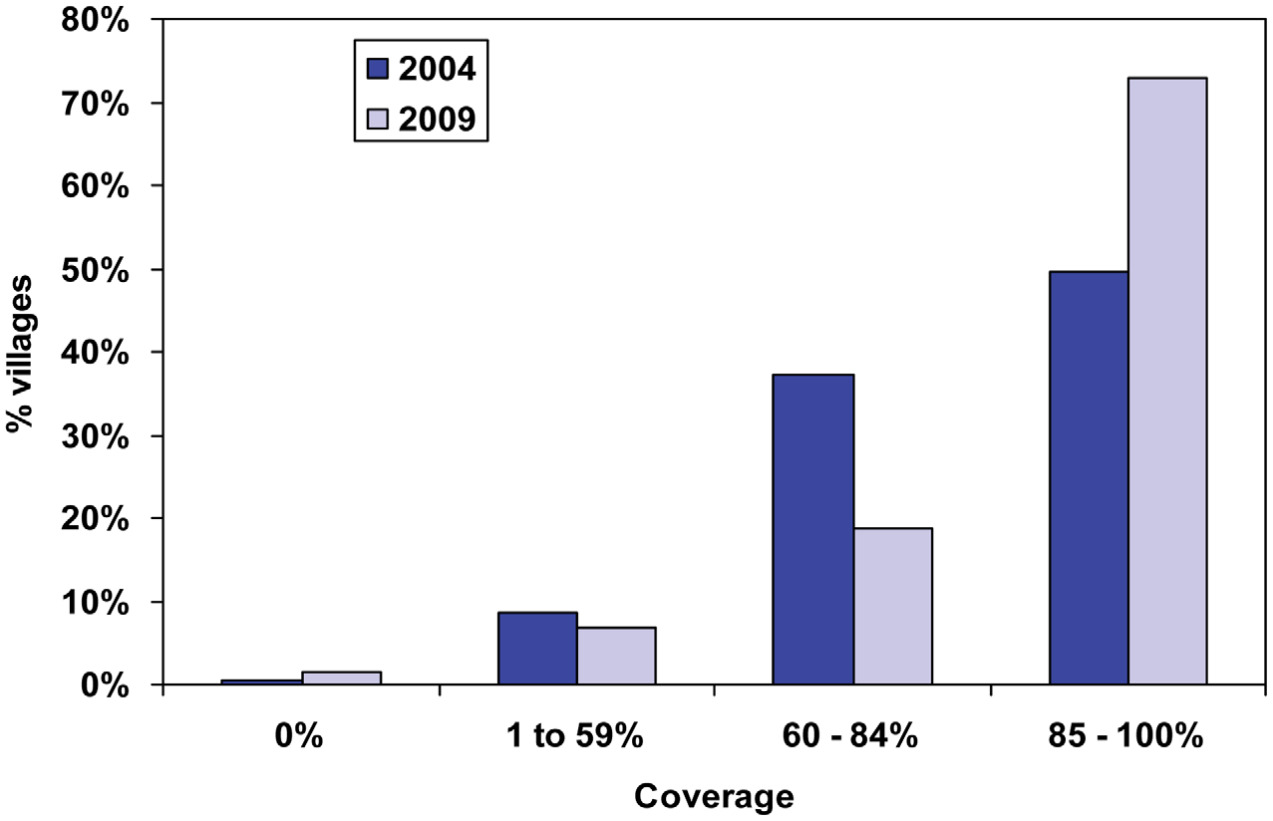
Reported village treatment coverage (eligible population): 2004 and 2009. This x axis shows reported village level treatment coverage ranges based on community CDD registers, and the y axis shows the percent of villages that reported coverages falling into that treatment coverage range for the years 2004 and 2009. Forty-nine percent of 3677 villages reported over 85% coverage of the eligible population in 2006, while 73% of 3638 villages reached that goal in 2009.

Scaling up to full geographic coverage was complete in 2003, four years after launching the program; 85% coverage of UTG was first reached in 2003, and then exceeded 87% for the next six years. Annual reported coverage in each of the ten SVs over this period (Table 1) showed a mean treatment coverage of 91% (range 50–100%). However, civil unrest prevented treatment in the Babale urban sentinel site in Jos East in 2009. Figure 3 shows reported village level treatment coverage, by coverage ranges, for the years 2004 and 2009. Forty-nine percent of 3,677 villages reported ≥85% coverage of the eligible population in 2006, while 73% of 3,638 villages reached that goal in 2009.

### Surveyed Treatment Coverage

The 2003 cluster coverage survey provided treatment figures for 4,524 treatment eligible persons (defined as age ≥5 years) drawn from a sample frame that included all 30 LGAs. Coverage was 72.2% (95% CI 65.5–79.0), and did not vary significantly between states. The 2003 urban coverage (n = 1,322) was 61.9% (CI 56.6–67). Surveyed coverage in the sentinel villages in 2003 was 82% (Table 1), which was significantly higher than that of entire two-state area, but lower than the 90% reported in SV treatment registers for that year.

### Surveillance for Adverse Events

Rates of adverse event reporting during the scale up period (2001–2003) ranged from 0.41–1.16%, and were lower (0.10–0.24%) in the years thereafter. This was as expected, since the most reactions are likely during the first rounds of therapy, when highest mf density carriers are present in the population. Events reported were also as expected, being primarily headache, fever and abdominal pain; there were no reports of scrotal pain or swelling noted in some LF areas treated with diethylcarbamazine (DEC)/albendazole MDA [38]. No life threatening reactions, or reactions requiring hospitalization, were reported during the ten year period.

### Monitoring the Impact in SVs

#### Nocturnal microfilaremia

**Figure 4:**
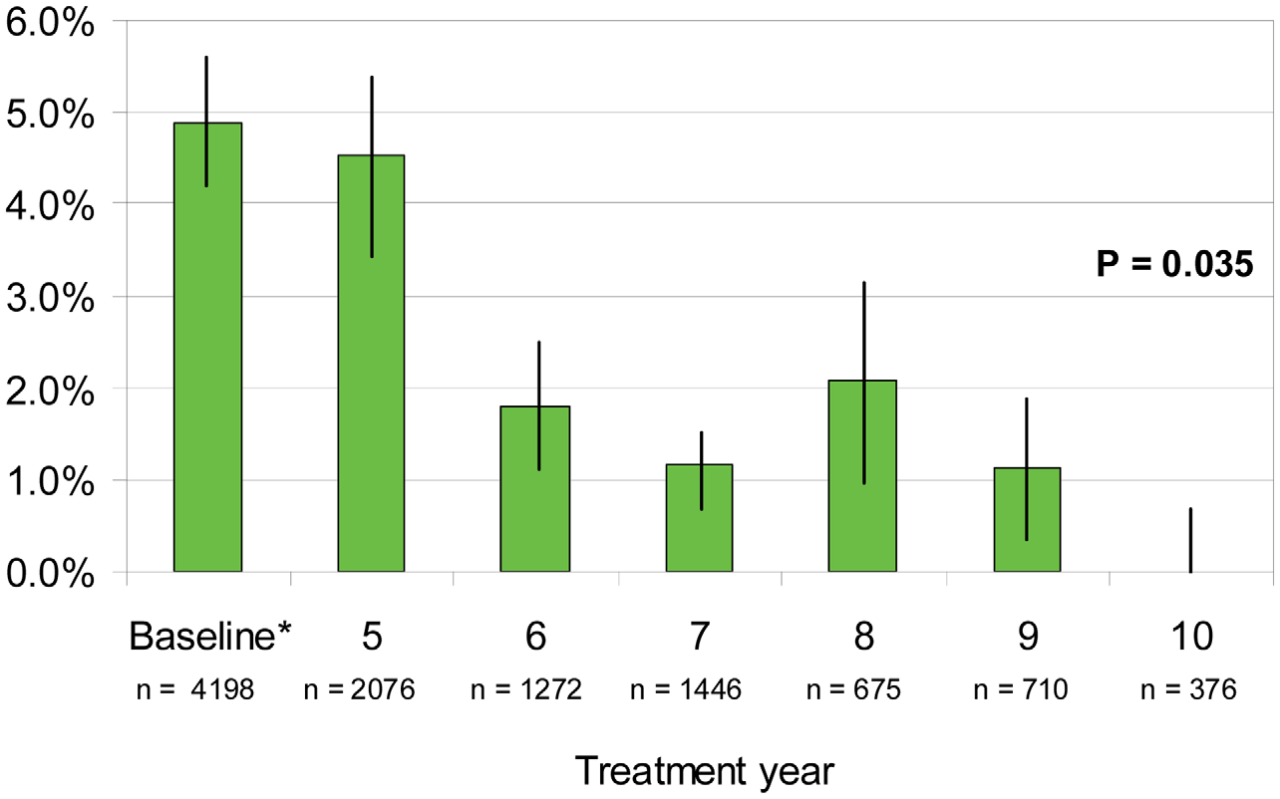
Mean sentinel village microfilaremia prevalence by MDA treatment year (n = 10,753). Nocturnal microfilaremia as determined by 60 ul thick smear. SV results across all four MDA phases have been adjusted to MDA treatment year for comparability. No pretreatment data are available for Gbuwhen, Gwamlar, Lankan, Maiganga and Seri, so earliest available mf data point was used as the baseline figure. Bars show 95% confidence intervals. Chi square for trend for all years was significant (p = 0.035), but was not significant using an analysis between baseline and MDA year 6 (p = 0.187). In addition, the key threshold of <1% microfilaremia was not attained by MDA year 6.

Achieving a nocturnal microfilaremia (mf) prevalence of <1% is the key WHO indicator of transmission for SVs. Mean SV mf prevalence (Table 2) dropped from a baseline of 4.9% to 0.9% in 2009 (p<0.01). In 2009 evaluations (10 months after the 2008 treatment), 7 of 9 SVs monitored had an mf prevalence under the 1% threshold. The two SVs with mf prevalence ≥1% were Gwamlar (4.9% after 8 years of MDA) and Piapung (2.1% after 7 years of MDA). However, recent annual fluctuations around that important threshold occurred for Seri, Maiganga, and Lankan. Mean mf prevalence adjusted by MDA year (Figure 4) decreased from 4.9% at baseline to 0% in year 10, but hovered above the critical 1% mark for all other years; after the sixth MDA the mean mf prevalence was 1.8%. The analysis for trend was significant (p = 0.035).

**Table 2:** MF prevalence in 10 sentinel villages: baseline and by year (n = 10,753).

			Baseline		2004		2005		2006		2007		2008		2009	
Sentinel Village	LGA	Years of Tx	% pos	n	% pos	n	% pos	n	% pos	n	% pos	n	% pos	n	% pos	n
**Akwete** ***	**Awe**	**7**	0.5%	424	NA	NA	NA	NA	NA	NA	0.0%	81	0.0%	45	0.0%	168
**Azara** ***	**Awe**	**7**	0.2%	402	NA	NA	NA	NA	NA	NA	0.0%	20	0.0%	61	0.0%	109
**Babale** ***	**Jos North**	**7**	0.4%	261	NA	NA	NA	NA	NA	NA	0.0%	96	0.0%	68	NA	NA
**Dokan Tofa** ***	**Shendan**	**7**	5.0%	419	NA	NA	NA	NA	NA	NA	1.3%	151	0.0%	158	0.4%	223
**Gbuwhen** °	**Akwanga**	**10**	3.7%	508	1.8%	446	0.3%	286	0.5%	183	0.0%	196	0.0%	127	0.0%	175
**Gwamlar** **	**Kanam**	**8**	6.7%	494	NA	NA	NA	NA	12.1%	240	1.6%	128	5.0%	100	4.9%	143
**Lankan** °	**Pankshin**	**10**	3.3%	274	7.4%	365	4.5%	243	2.5%	81	1.7%	117	4.0%	173	0.0%	201
**Maiganga** *	**Wamba**	**9**	4.7%	486	NA	NA	3.0%	169	5.6%	126	0.6%	158	1.8%	109	0.7%	152
**Piapung** ***	**Mikang**	**7**	9.9%	403	NA	NA	NA	NA	NA	NA	9.6%	187	NA	NA	2.1%	291
**Seri** *	**Kanke**	**9**	10.6%	527	NA	NA	1.6%	321	1.3%	157	0.8%	133	2.7%	110	0.0%	258
**TOTAL**			**4.9%**	**4,198**	**4.3%**	**811**	**2.2%**	**1,019**	**5.2%**	**787**	**2.1%**	**1,267**	**1.8%**	**951**	**0.9%**	**1,720**

Nocturnal microfilaremia as determined by 60 ul thick smear. The total n value in the table is also reflected in the related graph (Figure 4). Baseline data point is explained in Figure 4 legend. NA = not applicable or not available.

°Started treatment in 2000.

*Started treatment in 2001.

**Started treatment in 2002.

***Started treatment in 2003.

#### LF antigen

**Figure 5:**
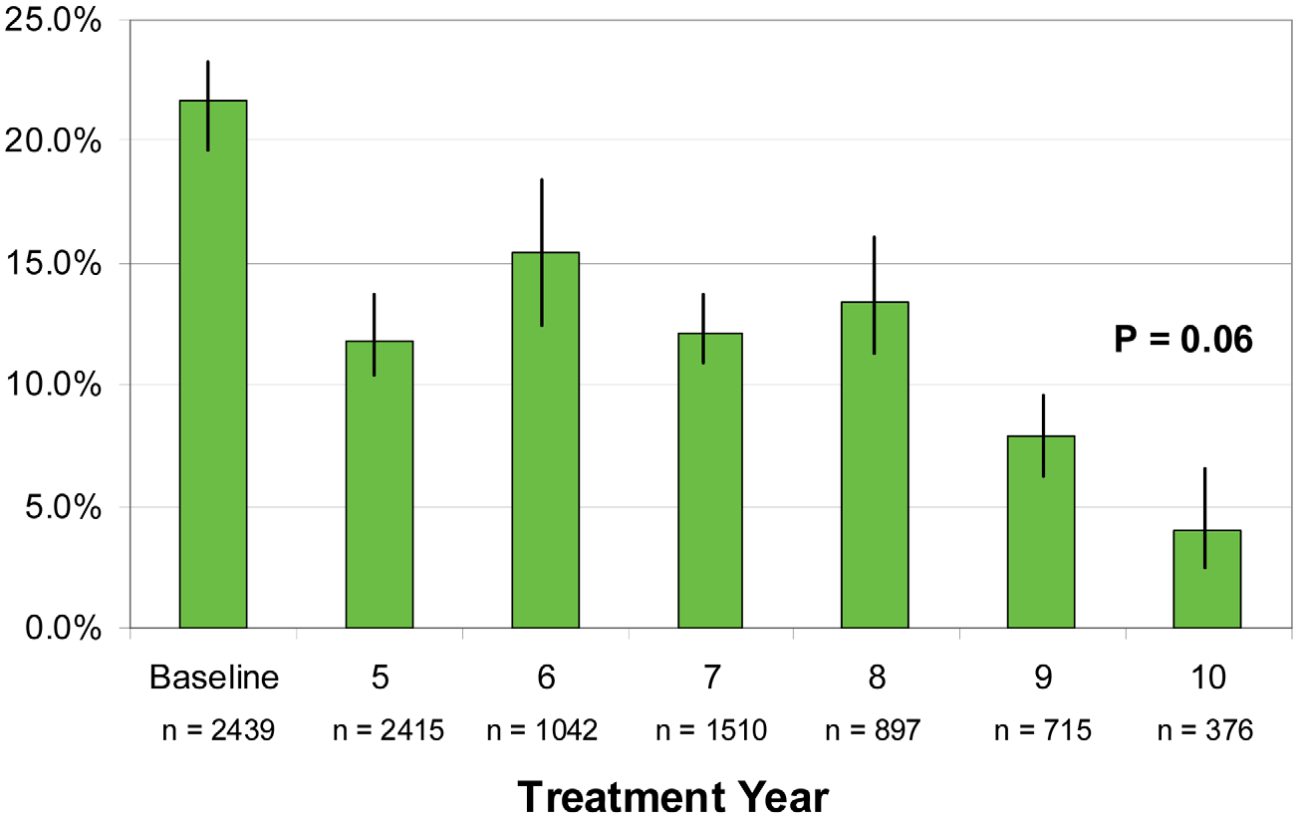
Mean sentinel village antigen prevalence by MDA treatment year (n = 9,394). Filarial antigenemia as determined by ICT testing. SV results across all four MDA phases have been adjusted to MDA treatment year for comparability. Data for the baseline antigen for five villages (Gbuwhen, Gwamlar, Lankan, Maiganga, and Seri) were from 1999–2000 mapping surveys. Baseline for the remaining villages (Akwete, Anzara, Babale, Dokan Tofa, Piapung) combined values from the community wide nocturnal pre-treatment surveys conducted in 2003 with pre-treatment data from the 1999–2000 mapping surveys. Chi square for trend not significant (p = 0.06 for all MDA years and p = 0.271 for baseline through MDA year 6). Bars show 95% confidence intervals.

The mean SV baseline antigen level (Table 3) of 21.6% dropped quickly to 10–15% due in large part to age sampling differences. Five SVs had 2009 antigen prevalence over 5%, with Gwamlar having a very remarkable 27.3% despite 8 years of MDA. When adjusted by MDA treatment year (Figure 5), a plateau is observed during years 5–8, resulting in an inability to demonstrate a significant trend over the period (p = 0.06). Testing between individual years show antigen levels after year 6 of MDA were significantly lower than baseline (p<0.01), even though year 6 antigen levels were still more than 50% of the baseline value (15.5% versus 21.6%).

**Table 3:** LF antigen prevalence in 10 sentinel villages: baseline and by year (n = 9,394).

			Baseline		2004		2005		2007		2008		2009	
Sentinel Village	LGA	Years of Tx	% pos	n	% pos	n	% pos	n	% pos	n	% pos	n	% pos	n
**Akwete** ***	**Awe**	**7**	13.5%	474	NA	NA	NA	NA	5.8%	191	6.6%	45	2.4%	168
**Azara** ***	**Awe**	**7**	9.3%	452	NA	NA	NA	NA	2.2%	185	4.9%	61	0.9%	109
**Babale** ***	**Jos North**	**7**	6.4%	311	NA	NA	NA	NA	1.5%	324	1.5%	68	unrest	NA
**Dokan Tofa** ***	**Shendan**	**7**	23.5%	469	NA	NA	NA	NA	14.4%	277	8.7%	158	3.0%	223
**Gbuwhen** °	**Akwanga**	**10**	46.7%	30	5.2%	446	4.5%	178	6.4%	282	0.0%	127	0.6%	175
**Gwamlar** **	**Kanam**	**8**	58.0%	50	NA	NA	NA	NA	31.5%	270	17.0%	100	27.3%	143
**Lankan** °	**Pankshin**	**10**	47.0%	100	23.6%	365	19.0%	200	20.6%	253	9.6%	178	7.0%	201
**Maiganga** *	**Wamba**	**9**	54.0%	50	NA	NA	20.6%	165	19.2%	234	1.8%	109	7.9%	152
**Piapung** ***	**Mikang**	**7**	31.8%	453	NA	NA	NA	NA	19.6%	312	14.5%	62	7.6%	291
**Seri** *	**Kanke**	**9**	62.0%	50	NA	NA	13.0%	150	22.6%	385	8.2%	110	10.5%	258
**TOTAL**			**21.6%**	**2,439**	**13.4%**	**811**	**14.4%**	**693**	**15.0%**	**2,713**	**7.4%**	**1,018**	**7.4%**	**1,720**

Filarial antigenemia as determined by ICT test. Baseline data point is explained in Figure 5 legend. The total n value in the table is also reflected in the related graph (Figure 5). NA = not applicable or not available.

°Started treatment in 2000.

*Started treatment in 2001.

**Started treatment in 2002.

***Started treatment in 2003.

#### Entomology

**Figure 6:**
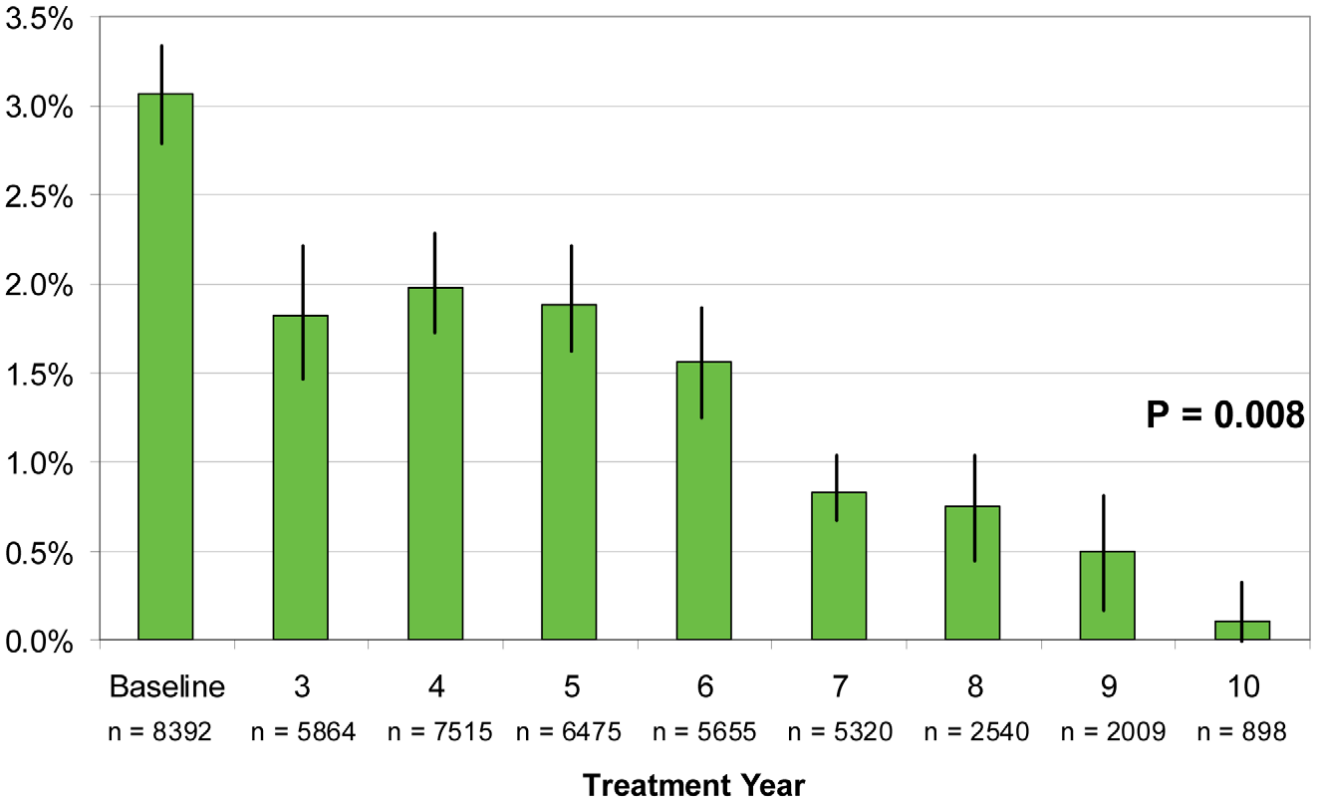
Mean mosquito infection (all larval stages) by MDA treatment year in 10 sentinel villages (n = 44,668). Dissections from bimonthly intradomiciliary pyrethrum knockdown collected mosquitos for all larval stages (L1–3) combined across all SVs and adjusted to MDA treatment year for comparability. Baseline mosquito infection rates are the aggregate values from pretreatment and the first two years of treatment; no baseline data were available for Babale SV. Chi square for trend for all years was highly significant (p = 0.008), but the trend analysis was not significant using data between baseline and MDA year 6 (p = 0.131). Bars show 95% confidence intervals.

Anopheline mosquitoes made up 96% of PK captures: 78% of captures were *Anopheles gambiae* s.l. and 18% *An. funestus*; *Culex* species were 3%. Only *Anopheles* sp mosquitoes were found infected with larvae of *W. bancrofti*. Table 4 shows baseline and annual infection rates, which were lowest in 2009 (0.4% of 4,398 dissections) and significantly reduced compared to baseline (p<0.01). Mosquito infection rates adjusted by treatment year (Figure 6) show a similar plateau to antigen levels during years 3–6, with an overall decrease from a 3.1% baseline to 0.1% in year 10. Infections were 1.6% in the sixth MDA year (significantly lower than baseline, p<0.01) and an overall highly significant trend analysis.

**Table 4:** Mosquito infection rates (all larval stages) in 10 sentinel villages: baseline and by year (n = 44,668).

			Baseline		2002		2003		2004		2005		2006		2007		2008		2009	
Sentinel Village	LGA	Years of Tx	% pos	n	% pos	n	% pos	n	% pos	n	% pos	n	% pos	n	% pos	n	% pos	n	% pos	n
**Akwete** ***	**Awe**	**7**	0.1%	1,660	NA	NA	NA	NA	NA	NA	0.0%	292	0.0%	721	0.4%	458	0.0%	355	0.0%	443
**Azara** ***	**Awe**	**7**	0.0%	164	NA	NA	NA	NA	NA	NA	NA	NA	0.0%	331	0.0%	130	NA	NA	NA	NA
**Babale** ***	**Jos North**	**7**	NA	0	NA	NA	NA	NA	NA	NA	NA	NA	0.0%	262	0.0%	250	0.0%	426	0.0%	262
**Dokan Tofa** ***	**Shendan**	**7**	2.4%	2,136	NA	NA	NA	NA	NA	NA	5.7%	897	4.7%	548	1.2%	402	1.2%	346	0.1%	684
**Gbuwhen** °	**Akwanga**	**10**	0.6%	798	0.00%	391	0.3%	1,840	0.5%	1,285	0.1%	1,143	0.2%	858	0.3%	732	0.2%	505	0.0%	569
**Gwamlar** **	**Kanam**	**8**	19.0%	105	NA	NA	NA	NA	NA	NA	NA	NA	13.4%	584	12.6%	587	9.3%	321	2.3%	352
**Lankan** °	**Pankshin**	**10**	5.9%	886	1.80%	389	1.0%	1,363	0.8%	1,395	0.0%	1,137	0.8%	745	0.6%	484	0.4%	224	0.3%	329
**Maiganga** *	**Wamba**	**9**	2.1%	885	NA	NA	0.5%	2,202	1.0%	1,203	0.9%	986	0.5%	976	0.1%	1,033	0.0%	583	0.3%	701
**Piapung** ***	**Mikang**	**7**	5.7%	871	NA	NA	NA	NA	NA	NA	2.1%	718	10.0%	768	0.5%	547	0.7%	306	0.2%	479
**Seri** *	**Kanke**	**9**	6.7%	887	NA	NA	2.5%	975	2.9%	479	1.6%	438	0.5%	379	0.8%	495	1.5%	389	1.0%	579
**TOTAL**			**3.1%**	**8,392**	**0.90%**	**780**	**0.7%**	**6,380**	**1.0%**	**4,362**	**1.5%**	**5,611**	**3.2%**	**6,172**	**1.8%**	**5,118**	**1.3%**	**3,455**	**0.4%**	**4,398**

Dissections from bimonthly intradomiciliary pyrethrum knockdown collections of mosquitoes showing percent infected with any larval stage (L1–3) determined by dissection. Results reflect only *Anopheles* sp mosquito dissections. Collections were generally performed in the same household compounds. Baseline data point is explained in Figure 6 legend. The total n value in the table is also reflected in the related graph (Figure 6). NA = not applicable, not available, or fewer than 100 mosquitoes dissected.

°Started treatment in 2000.

*Started treatment in 2001.

**Started treatment in 2002.

***Started treatment in 2003.

#### Phase 4 SVs

**Figure 7:**
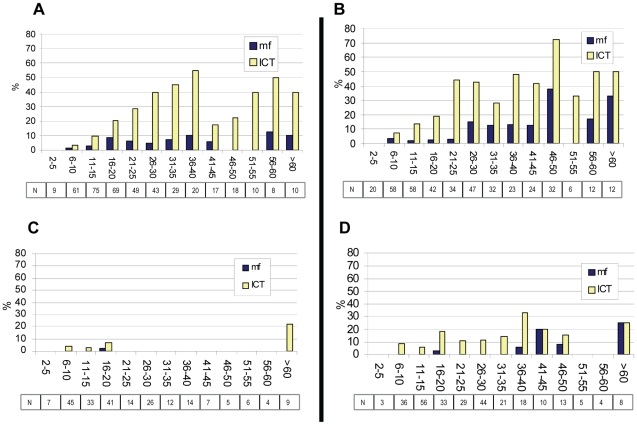
Age-specific prevalence for mf or LF antigen in Dokan Tofa and Piapung sentinel villages. Panel A: Dokan Tofa pretreatment prevalence by age group (n = 418). Panel B: Piapung pretreatment prevalence by age group (n = 400). Panel C: Dokan Tofa prevalence by age group after 6 rounds of MDA (n = 223). Panel D: Piapung prevalence by age group after 6 rounds of MDA (n = 280). Boxes show numbers sampled in each age group. Pretreatment sample for mf is different from Table 2 because ages were not available on all persons tested. Pretreatment sample for antigen for these SVs are different than baseline figure shown in Table 3 because 1999/2000 mapping data were added to baseline calculation in Table 3 (see Methods).

Phase 4 SVs were instructive because they were not pretreated with ivermectin monotherapy for onchocerciasis and they received LF MDA for the shortest period of time (six years). Piapung and Dokan Tofa were the most interesting because they had the highest pretreatment antigen and mf data of the Phase 4 SVs: Piapung had a baseline (pretreatment) mf prevalence of 9.9%, and antigen level of 31.8%; Dokan Tofa a baseline (pretreatment) mf prevalence of 5.0%, and antigen of 23.5%. Both SVs had interval reported treatment coverages and 2003 surveyed coverage of ≥85% (Table 1). Ten months after the sixth round of MDA, the change from baseline mf (Table 2) and antigen (Table 3) levels was statistically significant for both SVs (p<0.01). However, Piapung did not reach the <1% mf threshold, with a 2009 mf prevalence of 2.1% compared to Dokan Tofa (mf prevalence 0.4%). Age specific mf and antigen prevalence for these two SVs at baseline and 10 months after the Year 6 MDA treatment round are shown in Figure 7. Both villages show remarkable changes in both parameters in most age groups. In Piapung, which began with antigen levels representing the upper tercile (>25%), these changes are less dramatic. Both villages, however, showed a concerning number of 6–10 year old children with positive antigen tests, which could suggest ongoing transmission in both villages (Antigen positive children 5 years and under was not demonstrated at either baseline or follow-up). In entomological evaluations, an L3 (infective) mosquito was captured in Dokan Tofa in 2009.

#### Overall assessment of transmission based on SV studies

Table 5 is a summary table showing baseline and final (2009) measurements of mf, antigen, and mosquito infection and infectivity rates in the SVs. Average mf in the ten SVs decreased by 83%, from 4.9% to 0.8%, while the decrease of antigenemia was less marked at 67% (from 21.6% to 7.2%). Summary entomological data showed that the overall mosquito infection rate decreased by 86% (from 3.1% to 0.4%) and mosquito infectivity rate decreased by 76% (from 1.3% to 0.3%). All findings were highly statistically significant (p<0.01), as were many, but not all, individual SV decreases between baseline and 2009.

**Table 5:** Baseline and 2009 microfilaria, antigenemia and mosquito infection/infectivity in 10 SVs, and 2009 transmission status.

			Mf			Antigenemia			Mosquito Infection			Mosquito Infectivity (L3)			2009 Transmission
Sentinel Village	LGA	Years of Tx	Base	2009	%	Base	2009	%	Base	2009	%	Base	2009	%	
**Akwete**	**Awe**	**7**	0.5%	0.0%	100%	13.5%	2.4%*	82%	0.1%	0.0%	100%	0.0%	0.0%	N/A	Interrupted
**Azara**	**Awe**	**7**	0.2%	0.0%	100%	9.3%	0.9%*	90%	0.0%	0.0%	N/A	0.0%	0.0%	N/A	Interrupted
**Babale**	**Jos North**	**7**	0.4%	0.0%	100%	6.4%	1.5%	77%	NA	0.0%	N/A	NA	0.0%	N/A	Interrupted
**Dokan Tofa**	**Shendan**	**7**	5.0%	0.4%*	91%	23.5%	3.0%*	87%	2.4%	0.1%*	94%	1.0%	0.1%**	86%	Ongoing
**Gbuwhen**	**Akwanga**	**10**	3.7%	0.0%*	100%	46.7%	0.6%*	99%	0.6%	0.0%	100%	0.1%	0.0%	100%	Interrupted
**Gwamlar**	**Kanam**	**8**	6.7%	4.9%	27%	58.0%	27.3%*	53%	19.0%	2.3%*	88%	8.2%	0.9%*	90%	Ongoing
**Lankan**	**Pankshin**	**10**	3.3%	0.0%*	100%	47.0%	7.0%*	85%	5.9%	0.3%*	95%	2.3%	0.3%**	87%	Ongoing
**Maiganga**	**Wamba**	**9**	4.7%	0.7%**	86%	54.0%	7.9%*	85%	2.1%	0.3%**	87%	0.9%	0.0%**	100%	Interrupted
**Piapung**	**Mikang**	**7**	9.9%	2.1%*	79%	31.8%	7.6%*	76%	5.7%	0.2%*	96%	3.0%	0.0%*	100%	Ongoing
**Seri**	**Kanke**	**9**	10.6%	0.0%*	100%	62.0%	10.5%*	83%	6.7%	1.0%*	84%	2.0%	0.9%	57%	Ongoing
**TOTAL**			4.9%	0.8%*	83%	21.6%	7.2%*	67%	3.1%	0.4%*	86%	1.3%	0.3%*	76%	

% columns indicate percent decrease from baseline. 2009 Transmission status column (far right) indicates ‘interrupted’ only when Mf<1% and Mosquito Infectivity (L3) = 0. Babale final impact data points are from 2008 due to unrest in 2009. Azara mosquito infection final impact data point is from 2007 due to small sample sizes (<100) in 2008 and 2009.

*p<.01 compared to baseline.

**p<.05 compared to baseline.

The final column in Table 5 summarizes these findings into our conclusions as to whether transmission was interrupted based on 1) mf prevalence being <1% and/or 2) absence of L3 in mosquito dissections. We did not make judgments based on community-wide LF antigen results since most experts restrict assessments of antigen to younger age groups [7],[18],[39],[40]. If only the WHO SV standard of mf<1% were considered, then transmission interruption would have been judged to have occurred in 8 SVs, with the failures being Gwamlar (2009 mf prevalence of 4.9%) and Piapung (2009 mf = 2.1%). Using our entomological criterion (evidence of circulating L3 in vector mosquitoes), 4 of the 10 SVs (Gwamlar again, Dokan Tofa, Lankan, and Seri) had evidence of transmission. Taken together, therefore, we concluded that LF transmission had been interrupted in only 5 SVs. In this regard, considering initial force of transmission and its relationship to breaking transmission, it is useful to note that the successful SVs (with interrupted transmission) had baseline values that represented an average of 1.9% mf, 0.7% mosquito infection and 0.25% mosquito infectivity, compared to 7.1%, 7.9% and 3.3% respectively for baseline in SVs with ongoing transmission in 2009.

Six of the SVs had initial antigen levels that were >25% (the upper tercile cutoff in the mapping results. Four of the SVs were below this cutoff, with baseline antigen levels ranging from 6.4% to 23.5%. Of the six SVs with endemicity representing the upper tercile, four showed evidence of ongoing transmission in 2009. In contrast, only one of the four lower endemicity SVs in 2009 failed to interrupt transmission (due to a single infectious mosquito captured in Dokan Tofa). However, it should be noted that the other 3 SV (Akwete, Azara, and Babale), would have passed WHO SV transmission interruption criterion at baseline, before MDA was even launched (despite these SVs having baseline MDA antigen prevalences above the 1% launch MDA threshold that ranged from 6.4–13.5%). None of these SVs had been ‘pretreated’ with ivermectin for onchocerciasis.

## Discussion

We describe a large program scale up experience using annual combination ivermectin/albendazole mass drug administration (MDA) to interrupt anopheline transmitted *W. bancrofti* in two Nigerian states. To measure success, we serially monitored three infection parameters (microfilaremia, antigenemia, and mosquito infection) in 10 sentinel villages (SVs) scattered throughout the two state area. During the ten year SV monitoring period >10,000 nocturnal blood smears were read, >9,000 ICT tests performed, and >44,000 mosquitoes dissected. We found that the MDA program significantly reduced all overall infection parameters in 2009 compared to baseline. However, based on presence of microfilaremia ≥1% and/or the finding of L3 stages in mosquitoes, we believe that LF transmission continued uninterrupted in half of the SVs after six or more years of MDA. However, use of the WHO SV standard based on mf alone, 80% of the SVs were successful. In both cases, our findings are consistent with other reports noting that extended MDA intervention beyond six years may be required in some instances [13].

The success of the MDA strategy for LF rests on achieving and sustaining very high treatment coverage [7], [12], [13], [41]. Reported treatment coverage in Plateau and Nasarawa states was ≥85% of the 3.7 million treatment eligible persons aged ≥5 years (>77% of the total population of 4.1 million). However, population-based cluster surveys in 2003 (the first year of full geographic coverage) showed eligible population coverage of 72.2% (95% CI 65.5–79.0); below the 85% we sought. Discrepancies between reported and surveyed coverage have been previously noted by WHO LF technical committees [11]. We also surveyed the SVs in 2003, and found 82% of eligible persons reported taking the tablets, a figure below the reported SV coverage figures (90%) and above the 79% (upper 95% CI) determined by the state-wide population based survey. Thus, the impact results from the SVs represent the best case scenario for the two state area, but ambiguous (within the SVs themselves) with respect to the 85% treatment goal. An analysis of reported village coverage figures based on treatment registers kept by CDDs for 2009 showed that a quarter of villages failed to achieve the desired coverage. Program managers need to conduct analyses such as these to detect the subset of communities that require action to improve coverage [13], [15]. Further operations research in this area is needed.

The most important indicator in our SV monitoring activities was nocturnal microfilaremia. The WHO guidelines suggest SV monitoring of mf be conducted serially in each implementation unit during the MDA treatment period until a prevalence of <1% is reached. At that threshold, population based antigen surveys focused on children (called ‘transmission assessment surveys’—TAS) must be conducted to enable a full determination of transmission status and a ‘stop treatment’ decision for the implementation unit [7], [13], [18]. In Plateau and Nasarawa states, the costs and logistical challenges of monitoring 30 SVs (one in each LGA) were beyond the program's capacity [11]. Thus, we monitored only 10 SVs, but as a result we are unable to make individual LGA decisions based on SV data. The 83% decrease in mf compared to baseline is consistent with other reports [42]. Encouraged by these findings, and by the fact that 80% of SVs achieved the <1% goal, we elected to conduct a major ‘TAS-like’ population-based cluster survey to determine antigen prevalence for each LGA to decide where MDA could be stopped, and where MDA needed to continue. These results will be reported separately (King et al. in preparation).

The serial ICT testing in this study, while not required by WHO guidelines, provides insight into antigen decay in MDA programs based on ivermectin and albendazole. As expected, SV antigenemia decreased less dramatically than mf. The 2009 antigen prevalence remained above the 1% threshold used in 2000 to determine if MDA should be launched (8 of the ten SVs were above 1% antigenemia in 2009). It is likely that the drug combinations using ivermectin result in slower antigen decay than in MDA programs using DEC, which is more effective against the adult *W. bancrofi* worms [43]. Persistent antigenemia may not necessarily be an indicator of reproductively viable adult *W. bancrofti* worms that pose a threat of reestablishing transmission after MDA is withdrawn [8]. Therefore the importance of community wide antigen prevalence after many years of MDA is unclear, and not an indicator of treatment failure. Unlike mf, antigen decay should not fluctuate with the MDA treatment cycle, so antigen results are a reflection of the MDA year in which the test was performed. Therefore, our analysis suggests that antigen levels will remain >1% after ten years of MDA (Figure 5).

The SV entomology studies are relatively unique to the Plateau Nasarawa experience; such work is not a part of WHO guidelines [7], [18], [37]. Our dissections left us with a bleaker impression of impact than if we had used microfilaremia as the sole transmission criterion. Mosquito infectivity rates (L3 stages) provided immediate (‘real time’) evidence for community LF transmission activity [44], [45]. Based on bimonthly monitoring of mosquito infection in selected compounds, the average 2009 infection rate decreased significantly compared to baseline by 86% (from 3.1% to 0.4%); and infectivity rate (L3 stages) by 76% (from 1.3% to 0.3%). Using a finding of a single L3 as evidence of active transmission, we reclassified 3 SVs from ‘transmission interrupted’ (based on having microfilaremia <1%) to ‘transmission ongoing’ (Table 5). Models suggest that some residual L3 may circulate even after the transmission breakpoint has been reached (e.g., when *W. bancrofti* population is in irreversible decline) [12], [41], so our use of this stringent entomologic criterion divorced from entomological inoculation rates or transmission potential calculations could be challenged [44], [45], [46].

The MDA program took four years to scale up to full geographic coverage. As a result, by 2009, different LGAs had different MDA exposure histories. To better understand the annual impact of the treatment program we adjusted for these LGA differences by combining SV data by their MDA treatment year (Figures 4, 5, and 6). The MDA year analysis suggests that, had full geographic coverage been obtained immediately in all LGAs, ten years of MDA would be required to achieve mf <1% in all SVs; even at ten years 4% antigenemia and low grade mosquito infection (including L3—Table 5: Pankshin) might still be found. WHO technical committees continue to grapple with this problem [11], [13].

This analysis is complicated by the fact that the LF program was launched within onchocerciasis LGAs by first piggybacking onto the onchocerciasis MDA logistics. While this made programmatic sense, epidemiologically it meant that the LGAs likely to need the least MDA treatment (having had six or more years of ivermectin monotherapy for onchocerciasis) were the same ones to benefit from the longest treatment with the ivermectin and albendazole combination. The ivermectin ‘naïve’ LGAs of Phases 3 and 4 had considerably shorter drug experiences, and the SVs in these LGAs were the most instructive in terms of the WHO six year MDA treatment policy. Most important among these were Dokan Tofa and Piapung because they had the highest baseline mf and antigen prevalences of Phases 3–4 SVs. By 2009, the MDA program had significantly decreased mf, antigen and mosquito infection rates in Dokan Tofa and Piapung (Table 5), yet both SVs failed to meet our criteria for interrupted transmission: Piapung had microfilaremia above 1% , and Dokan Tofa (while mf were <1%) was found to have an infective mosquito. Piapung, the only one of the two that failed the WHO SV criteria (mf<1%), began treatment in the mapping upper tercile antigen category of >25%.

One SV (Gwamlar) was an MDA treatment failure: after 8 years of dual MDA, and earlier ivermectin monotherapy for onchocerciasis, 2009 monitoring showed microfilaremia of 4.9%, statistically unchanged from the baseline of 6.7%. This may have been because of frequent insecurity in the Kanam LGA, where Gwamlar is located. It was also only in Gwamlar that we accepted a ‘baseline’ mf data point in the sixth year of treatment (2005). There could have been a considerable drop in mf from an earlier (unmeasured) point in time. However, antigen prevalence in 2009 was highest of all SVs (27.3%) and mosquito infection rates >2% (with L3 found). Gwamlar's reported coverage figures were acceptable (interval mean of 86% – Table 1), but it was the one SV where the 2003 coverage survey was not conducted, due to insecurity. A detailed reassessment and increased interventional effort are needed there. Similar SV MDA treatment failures have been reported in Burkina Faso [13] and Ghana [42].

Each of the 30 LGAs was considered as a separate ‘implementation unit,’ to be ultimately judged separately with respect to LF transmission status and the decision to continue or halt the MDA intervention. Based on the initial mapping results, the upper tercile of ten LGAs showed an antigen prevalence of >25%. We assumed that transmission would be most difficult to break in those LGAs. We can test that hypothesis by comparing SV success at interrupting transmission by whether they had antigen levels >25% at baseline. We found that 4 of the 6 SVs having baseline antigen levels of >25% failed to interrupt transmission, compared to 1 of 4 SVs with baseline antigen ≤25%. Gwamlar's LGA, Kanam, had the second highest baseline antigen results in the mapping survey (44%), superseded only by Kanke LGA (baseline antigen 51%), whose SV (Seri) achieved <1% mf but not the absent L3 threshold. These observations support the idea that LGAs with greater endemicity (in this case those exceeding 25% antigen prevalence shown in red in the map in Figure 1) are likely to be the primary areas of risk of MDA failure in Plateau and Nasarawa states beyond 2009. It also appears to support the principle that an eradication or elimination program should seek to intervene as early as possible in the highest endemic areas, since they will require the longest time to interrupt transmission.

A change in activities to meet the challenges of the ‘end-game’ for this LF MDA program now needs to be considered [11]. Our data suggest that in some LGAs the MDA program could be halted, while in others six or more years of annual MDA alone has failed to interrupt transmission. Additional interventions are needed there, such as twice per year treatment, [47], [48] and/or insecticide treated bednets [12], [46], [49], [50]. The 2010/2011 distribution of long lasting insecticidal nets (LLIN) throughout Plateau and Nasarawa states by the national malaria program (with of goal of providing 2 LLIN per household) is a welcome addition to LF interventions.

There were several weaknesses to the monitoring program. First, SV coverage figures fall into the highest range of coverage values, and so SV impact results are not likely to be representative of all villages in the two-state area. Second, at least one additional coverage survey after 2003 would have been useful to compare with reported coverage results, especially given the increase in CDD numbers that occurred after that year. Third, building an experienced and trained monitoring team took time, and we were hampered by laboratory problems with Giemsa staining of blood slides (for the all-important mf monitoring) in the first years of the program, resulting in the loss of hundreds of baseline mf slides. Fourth, SV residents became tired of the annual nocturnal blood surveys and participation rates dropped over the course of the monitoring process. An example of the change in the representativeness of the SV samples can be seen in the 60% drop in participation rates between 2003 and 2009 nocturnal surveys in Dokan Tofa and Piapung SVs (Figure 7). Future nocturnal SV evaluations should be done less frequently, and not at all in the first 3 years of the MDA program. SV work should be staggered so that communities do not become fatigued, but evaluation teams do not lose their hard-earned field and laboratory skills. When the Nigerian LF program goes to scale, the most experienced technical teams from mature LF program states should move from state to state to provide training and oversight in the monitoring activities. Fifth, we did not count microfilaria on blood slides, nor did we record numbers of L1, L2 and L3 larvae found in positive mosquitoes. This prevented an analysis of density of *W. bancrofti* infection in the human (the community microfilarial load—CMFL) and vector population. As a result our datasets will not be particularly useful for mathematical disease modeling of the LF transmission system, as determination of force of transmission is difficult to approach using prevalence data alone.

The program met a number of challenges successfully. First was the challenge of logistics. Plateau and Nasarawa states have a combined population of over 4 million, comparable to that of a small country. Distances to be traveled were great and many villages were in very remote and difficult to access areas. Large numbers of ivermectin and albendazole tablets shipped from two different pharmaceutical companies at different times and entering the country in different ways had to be coordinated to provide both medicines to the same mouths at the same time. In the early years, ivermectin for LF was ordered and imported differently from ivermectin for onchocerciasis, meaning there were three drug shipments for two drugs to be coordinated: albendazole shipments for LF, ivermectin shipments for LF, and ivermectin shipments for onchocerciasis (for the 12 LGAs that happened to be co-endemic for LF). Another logistical problem was that of financing, ordering, importing, and storing the ICT tests. ICTs were not available in 2001 and 2006 due to production shortages. The manufacturer changed four times between 1998 and 2009, and with it the required training for new card configurations. Prices increased rather than decreased over the course of the program. The need to refrigerate later versions of the tests meant considerable added costs of purchasing refrigeration units, running generators (during power outages), use of cold boxes to transfer tests to the field, etc. Shortened shelf life of the tests to 3 months [11], [13] made multiple shipments in a year necessary. Logistics were also complicated by the challenges of frequent government strikes by state and LGA staff. The importance of having a focused NGO partner working closely with the state and LGA MOHs helped overcome what might have been insurmountable logistical and continuity issues.

Second was the challenge of insecurity, an ongoing concern in Plateau and Nasarawa states. The presence of village-based local resident distributors generally resulted in maintenance of treatment coverage even when LMOH or SMOH teams had to restrict their travel. For example in 2002 broad civil and religious unrest challenged the program in 8 of the 30 LGAs (Bassa, Kanam, Wase, Langtang North, Riyom, Doma, Keana, and Obi), but treatments in those LGAs were not affected (data not shown). On only one occasion, due to 2009 rioting in the city of Jos, Jos East LGA (with its urban SV of Babale) could not be treated.

A new experience in 2003 was executing the MDA program in urban environments. Up until then, program managers had only worked in rural villages [35]. They learned very quickly that treatment in urban areas could not rely on community volunteers and traditional leadership structures. The new strategy was to engage organized religious groups to mobilize community participation and individual volunteerism: churches and mosques were approached in neighborhoods where the preponderance of the population was Christian or Moslem respectively. HE activities were held largely on Fridays (Moslems) or Sundays (Christians). Another urban innovation was the extensive use of TV and radio LF ‘jingles,’ aired constantly in Hausa and English during the campaign period. Rather than house to house, treatments were organized in central locations that served as distribution posts, commonly near a neighborhood church, mosque, health clinic or hospital. Nurses in white uniforms or white coats were important for these urban dwellers. While most treatment was still directly observed, sometimes treatments had to be given to family members to take home to absentees. Such practices were by and large unheard of in rural MDA activities. The 2003 coverage survey suggested that coverage in urban areas (61.9%, CI 56.6–67.0) was less than overall coverage (72.2%, 95% CI 65.5–79.0), but not significantly so.

From the onset, the program was committed to the concept of developing an integrated model for NTD activities [26]. The LF experience cannot be divorced from the longer standing onchocerciasis efforts in Plateau and Nasarawa states. First there was the important question of whether MDA for LF was even necessary in the 12 LGAs where ivermectin monotherapy MDA for onchocerciasis had been given for many years. Had there been a ‘by-stander’ effect where onchocerciasis MDA had inadvertently interrupted LF transmission? Our entomological, antigen and patient studies concluded that (unfortunately) LF transmission and morbidity persisted in such onchocerciasis monotherapy areas [27], [28], [30]. Following that conclusion, we initiated the LF MDA program by building upon the existing ivermectin delivery platform, where both programs would use the same drug (ivermectin) in the same LGAs. At the time, when integration was not as fashionable as it is today, many LF experts criticized launching the pilot LF program in an integrated fashion with onchocerciasis, arguing that the first LF programs ought to be launched in ivermectin naïve areas (our Phases 3 and 4) to enable ‘valid’ baseline (e.g., pretreatment) assessments of mf in SVs and better ‘roll out’ monitoring of adverse events. Tension between the LF program (seeking speed to reaching full geographic coverage for LF elimination within six years) and the onchocerciasis program (seeking 15–20 year sustainability of ivermectin distribution for onchocerciasis control) arose, particularly with respect to selection and training of CDDs. The benefits of piggybacking the LF program on the river blindness platform, however, were lost by the third year of the program, as the LF program moved into the ‘non onchocerciasis’ LGAs of Phases 3 and 4. The LF program had to rapidly scale up new community distribution networks to expand the MDA population under treatment by over 300%. By the end of 2003, recruitment and training of CDDs increased by 3 fold compared to 2000, from 2,424 to 6,899. In attempts to improve coverage and minimize workload further, the program continued to increase CDD numbers annually, by training over 10,000 in 2009. Increased CDD numbers was associated with improved village coverage, with the percentage of villages achieving ≥85% coverage increasing from 50% in 2006 to 73% in 2009.

As the LF program in Plateau and Nasarawa seeks to finance and undertake additional assessments to determine when and where MDA can be halted, considerations about the future of the onchocerciasis control program will arise. Consider the two options for MDA program adjustment within the 12 LF onchocerciasis co-endemic LGAs if it is decided that LF transmission has been broken: Option 1) Stop albendazole but carry on with ivermectin MDA for onchocerciasis, while conducting post treatment surveillance for LF recrudescence (recrudescence would be less likely to occur in the presence of ongoing ivermectin monotherapy compared to non onchocerciasis endemic LGAs where both ivermectin and albendazole would be stopped). Option 2) Determine the status of onchocerciasis transmission, with the thought of stopping treatment for onchocerciasis as well if indicated. Recent studies in parts of Mali and Senegal have shown that onchocerciasis transmission has been interrupted by 15 or more years of ivermectin treatment, and MDA can be safely withdrawn [51]. If community wide MDA treatments for LF and onchocerciasis can be stopped simultaneously in qualifying LGAs of Plateau and Nasarawa, then surveillance teams can make the best of scarce resources by undertaking ‘integrated’ post-treatment surveillance monitoring for both conditions. In the interest of controlling soil transmitted helminths (STH) and schistosomiasis, either scenario above would potentially still require MDA with albendazole and/or praziquantel in school aged children [52], [53].

Managing these MDA transitions in Plateau and Nasarawa states will be the final challenge. The complexity of modifying programs will depend on the mosaic of epidemiological findings likely to become more obvious in the near future as more assessments are undertaken. Some LGAs (mostly likely those with higher endemicity or poorer coverage) will require ‘mop up’ and enhanced interventions and operations. Other LGAs might move to post treatment surveillance activities. Individualized and tailored programmatic processes must be resolved eventually by the LGA level leadership and resources, where the health system structures on ground need the greatest support.
